# Inhibition of inflammation but not ankylosis by glucocorticoids in mice: further evidence for the entheseal stress hypothesis

**DOI:** 10.1186/ar3772

**Published:** 2012-03-12

**Authors:** Kirsten Braem, Christophe M Deroose, Frank P Luyten, Rik J Lories

**Affiliations:** 1 Laboratory for Skeletal Development and Joint Disorders, Department of Development and Regeneration, KU Leuven, Herestraat 49, Leuven, 3000, Belgium; 2 Division of Rheumatology, University Hospitals Leuven, Herestraat 49, Leuven, 3000, Belgium; 3 Division of Nuclear Medicine, University Hospitals and KU Leuven, Herestraat 49, Leuven, 3000, Belgium

## Abstract

**Introduction:**

Studies in the spontaneous ankylosis model in aging male DBA/1 mice and in patients with ankylosing spondylitis provide evidence that inflammation and new tissue formation leading to joint or spine ankylosis are likely linked but largely uncoupled processes. We previously proposed the 'entheseal stress' hypothesis that defines microdamage or cell stress in the enthesis as a trigger for these disease processes. Here, we further investigated the relationship between inflammation and ankylosis by focusing on the early phase of the spontaneous arthritis model.

**Methods:**

Aging male DBA/1 mice from different litters were caged together at the age of ten weeks and studied for signs of arthritis. A group of DBA/1 mice were treated daily with dexamethasone (0.5 μg/g body weight). Severity of disease was assessed by histomorphology and by positron emission tomography (PET) using 2-[^18^F]fluoro-2-deoxy-D-glucose (^18^F-FDG) as a tracer. Bone loss in dexamethasone-treated or control mice was determined by *in vivo *dual-energy X-ray absorptiometry. Chemokine gene expression was studied *ex vivo *in dissected paws and *in vitro *in mesenchymal cells (periosteal and bone marrow stromal cells) by quantitative real-time PCR in the presence or absence of bone morphogenetic protein 2 (BMP2) and dexamethasone.

**Results:**

Dexamethasone treatment did not affect incidence or severity of ankylosis, but led to an expected reduction in inflammation in the paws at week 15 as measured by PET tracer uptake. Treatment with dexamethasone negatively affected bone mineral density. Chemokines attracting neutrophils and lymphocytes were expressed in affected paws. *In vitro*, BMP2 stimulation upregulated chemokines in different mesenchymal joint-associated cell types, an effect that was inhibited by dexamethasone.

**Conclusions:**

BMP signaling may be a trigger for both inflammation and ankylosis in the spontaneous model of ankylosing enthesitis. The lack of inhibition by glucocorticoids on new bone formation while causing systemic bone loss highlights the paradoxical simultaneous loss and gain of bone in patients with ankylosing spondylitis.

## Introduction

Ankylosing spondylitis (AS) and related spondyloarthritides (SpA) are common chronic inflammatory joint diseases with severe impact on patients and society [1,2]. Inflammation is held responsible for many of the signs and symptoms of disease but the long-term prognosis for patients with AS is also determined by progressive ankylosis of the spine due to new cartilage and bone formation [3]. Remarkably, new bone formation at the bone borders occurs simultaneously with inflammation-induced loss of trabecular bone leading to osteoporosis [4,5].

Although traditionally viewed as a repair response, we hypothesized that ankylosis is a specific and primary aspect of AS and proposed the entheseal stress hypothesis which explains how inflammation and ankylosis are linked but largely uncoupled processes [6]. In this concept, microdamage or cell stress in the enthesis triggers both an inflammatory and a bone anabolic response leading to the clinical development of AS and the typical radiographic signs of disease. Specific environmental and genetic factors are suggested to influence chronicity of the inflammatory response and progression of ankylosis [6].

The introduction of anti-tumor necrosis factor (TNF) strategies has been a breakthrough for patients with AS and other SpAs [2]. These drugs have an unprecedented effect on symptoms of disease. However, recent data in mice and men suggest that control of inflammation with TNF blocking agents is not sufficient to prevent progressive ankylosis [7-10]. Therefore, in contrast to what is seen in patients with rheumatoid arthritis, anti-TNF appears to fail to inhibit radiographic progression of disease in AS [11,12].

DBA/1 mice spontaneously develop arthritis of the hind paws characterized by entheseal ankylosis upon grouped caging of males from different litters [13]. In this mouse model both anti-TNF treatment using human soluble receptor etanercept and anti-osteoclast strategies using zoledronic acid are not sufficient to inhibit new bone formation [7,14]. In contrast, overexpression of noggin, a bone morphogenetic protein (BMP) antagonist, reduced the incidence and severity of the murine arthritis [15].

The specific role of inflammation in this model remains unclear. We have demonstrated a short-lived inflammatory phase characterized by edema and neutrophil infiltration in the affected toes, likely preceding the remodeling phase [13]. Here we focused on these early inflammatory events, the interactions between BMPs and inflammation and the complex effects of glucocorticoids, drugs with strong anti-inflammatory and anti-bone anabolic effects, in the spontaneous model.

## Materials and methods

### Animal experiments

Male DBA/1 mice were obtained from Janvier (Le Genest St Isle, France). All experiments were approved by the Ethics Committee for Animal Research (KU Leuven, Belgium). Male mice from different litters were mixed and caged in groups of six mice at the age of ten weeks. Mice were treated daily with dexamethasone (0.5 mg/kg; Rotexmedica, Trittau, Germany) or phosphate buffered saline (PBS) by intraperitoneal injection from the age of 12 weeks onwards (*n *= 10 and 12 mice per group, respectively). Mice were scored blindly twice a week for clinical signs of arthritis [13,16]: 0 (no symptoms), 1 (redness and swelling in one toe), 2 (redness and swelling in more than one toe), 3 (toe stiffness), and 4 (deformity or ankle involvement). Hind paw forefeet were studied by histomorphology as described [13,16]: 0 (normal toe), 1 (acute inflammation including dactylitis), 2 (entheseal cell proliferation), 3 (cartilage formation), 4 (bone formation), and 5 (joint ankylosis). A cumulative score from all toes was calculated. In additional experiments, paws of control mice of 12 and 16-week-old DBA/1 mice were used for gene expression analysis.

### Imaging studies: small animal Positron Emission Tomography (PET) imaging and Dual Energy X-ray Absorptiometry (DEXA)

The mice were imaged using a Focus 220 microPET^® ^scanner (Concorde Microsystems Inc., Knoxville, TN, USA). The mice were anesthetized with 2.5% isoflurane in 100% oxygen at a flow rate of 1 L/minute and 7.4 MBq of 2-[^18^F]fluoro-2-deoxy-D-glucose (^18^F-FDG) was administered by lateral tail vein injection. One hour after injection a ten minute static scan was acquired. Images were reconstructed using a maximum *a posteriori *algorithm from the vendor with 18 iterations and uniform resolution. The images were displayed in Amide's a Medical Imaging Data Examiner (AMIDE) [17] and three-dimensional spheroid regions of interest (ROIs) were drawn over the toes, the paws and within a reference region (liver). The mean radioactivity in the ROIs was converted to percent of the injected dose per gram of tissue (%ID/g) by a calibrated cylinder factor and after division with the injected dose (corrected for residual and decay). Standardized uptake values (SUV) were obtained by multiplying the % ID/g of the toe area by the weight of the animal.

Bone mineral content was assessed by dual X-ray absorptiometry using Piximus densitometer (Lunar, Madison, WI, USA) [18].

### Cell culture

Human periosteum-derived cells (hPDCs) were isolated as described [19]. Human bone marrow cells (hBMCs) were isolated by standard aspiration from freshly isolated bone marrow as described previously [20]. hPDCs and hBMCs were expanded in complete culture medium consisting of high-glucose Dulbecco's Modified Eagle Medium (DMEM)/Glutamax (4.5 g/l of glucose) (Invitrogen, Merelbeke, Belgium) supplemented with 10% fetal bovine serum (Gibco, Merelbeke, Belgium), antibiotic-antimycotic solution (100 units/ml penicillin, 100 μg/ml streptomycin and 0.25 μg/ml amphotericin B; Invitrogen) and sodium pyruvate (Gibco). hPDCs and hBMCs were seeded at a density of 12 × 10^4 ^c/ml in 24-well plates. After overnight adherence, cells were stimulated with 3 to 30 ng/ml BMP2 (R&D Systems, Minneapolis, MN, USA) with or without 1 μM dexamethasone (Rotexmedica). After a 24-hour incubation, samples were collected for RNA analysis.

### RNA isolation and Real-Time RT-polymerase chain reaction (PCR)

For gene expression analysis of mouse tissues, hind paws of 12 and 16 week old mice were dissected just above the ankle joint and skin was removed. Total RNA was isolated using the standard Nucleospin RNAII kit (Macherey-Nagel, Düren, Germany) protocol. Complementary DNA was obtained by reverse transcription of 0.5 to 1 μg of total RNA using the RevertAid H Minus First Strand cDNA synthesis kit (Fermentas, Rockford, IL USA) following the manufacturer's instructions. For quantitative analysis, real-time RT-PCR was performed using Perfecta qPCR FastMix (Quanta Biosciences, Inc., Gaithersburg, MD, USA) carried out on a Corbett Rotor-Gene 6000 system (Corbett Research, Westburg, Leusden, The Netherlands). The expression of the following genes was examined: human chemokine (C-X-C motif) ligand 1 (*CXCL1*), granulocyte chemotactic protein 2 (*GCP2*), and inhibitor of DNA binding 1 (*ID1*) and mouse homolog *Cxcl1 *and *Gcp2 *and mouse chemokine (C-X-C motif) receptor 1 *(Cxcr1) *and *Cxcr2*. Human and mouse glyceraldehyde-3-phosphate dehydrogenase (*GAPDH *and *Gapdh*, respectively) were used as housekeeping genes. Primer/probe sets for these genes were commercially available Assay-on-demands (Applied Biosystems, Lennik, Belgium).

### Statistical analysis

For comparison between groups, Student's t-test was used. Results were considered statistically significant with two-sided *P*-values < 0.05.

## Results

### Severity of arthritis in mice treated with dexamethasone

**Figure 1:**
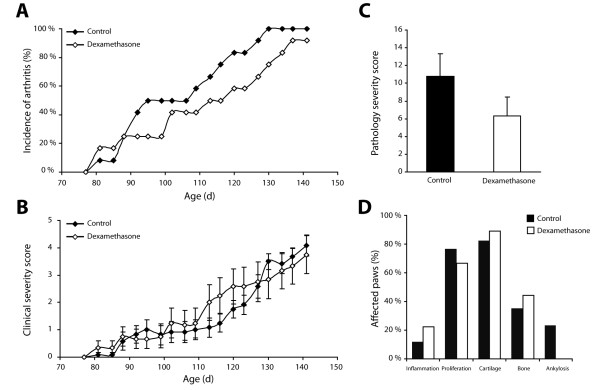
**Corticosteroid treatment does not affect clinical signs of spontaneous arthritis**. (**A**) Cumulative incidence of arthritis and (**B**) clinical severity score in dexamethasone-treated and control animals (*n *= 10 and 12 animals per group, respectively). (**C**) Dexamethasone treatment does not affect histological severity of arthritis (ns: *P *= 0.2). Data in (**B**) and (**C**) are shown as mean ± SEM. (**D**) Effect of dexamethasone treatment on different features of ankylosing enthesitis. Data in (**D**) are shown as percentage of affected paws exhibiting a specific treat compared to the total number of affected paws. ns, nonsignificant; SEM, standard error of the mean.

Daily injection of dexamethasone (0.5 mg/kg) from week 12 onwards did not inhibit clinical incidence or severity of spontaneous arthritis in male DBA/1 mice as compared to placebo-treated mice (Figure 1A-B). As the clinical evaluation of this model can not distinguish inflammatory and remodeling features, histomorphological analysis was performed. We found no difference in overall severity between dexamethasone-treated and control mice (Figure 1C). In addition, there was no significant difference in pathological endochondral bone formation between both groups (Figure 1D).

**Figure 2:**
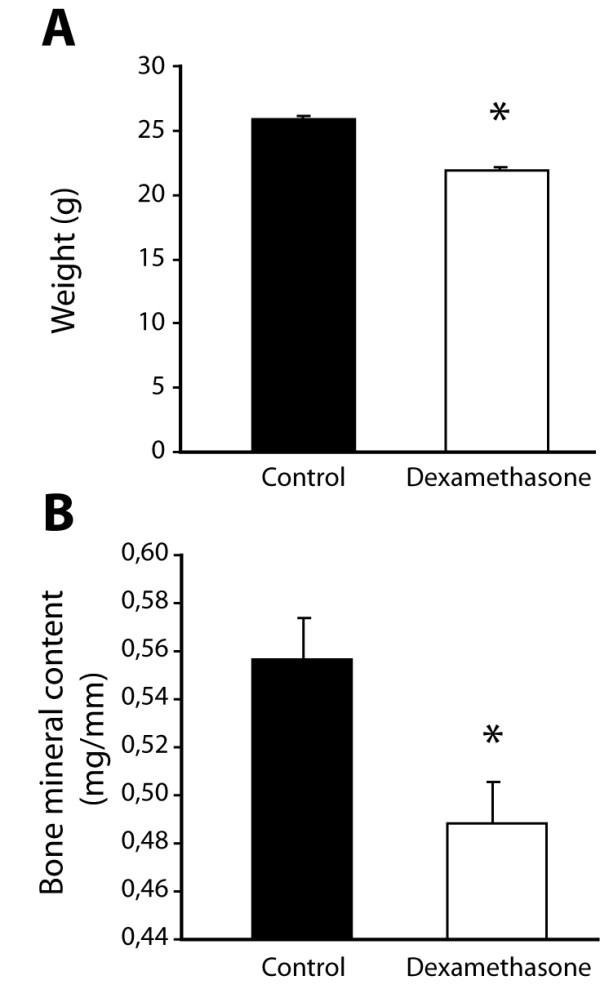
**Dexamethasone treatment affects body weight and whole body mineral content**. (**A**) Body weights were measured after two weeks of dexamethasone treatment. (**B**) Bone mineral content was measured by DEXA at the end of the experiment (20 weeks). Data are shown as mean + SEM. (*n *= 12 control animals and 10 dexamethasone-treated animals; * *P *< 0.05). DEXA, dual energy X-ray absorptiometry; SEM, standard error of the mean.

As expected dexamethasone treatment did significantly affect mouse weight (Figure 2A) and total body bone mineral content (Figure 2B), demonstrating that local new bone formation leading to joint ankylosis can occur simultaneously with systemic bone loss.

### Control of inflammation by glucocorticoid treatment

**Figure 3:**
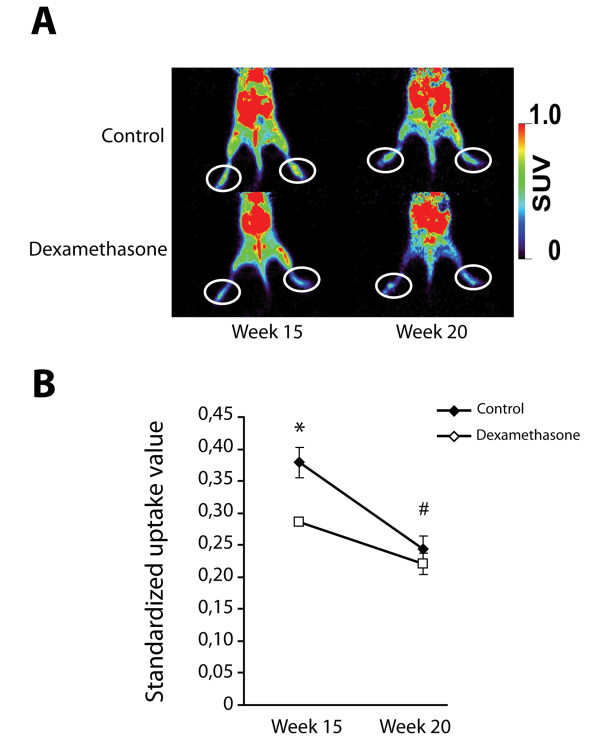
**Dexamethasone treatment influences the inflammatory component of spontaneous arthritis as measured by ^18^F-FDG PET scan**. (**A**) Representative images of animals from both treatment groups. The 'toe' area used for measurements is indicated. (**B**) Standardized uptake values of the tracer demonstrating a reduction in the uptake after dexamethasone treatment at week 15. At week 20, the inflammatory component has largely disappeared as seen in the control group. (*n *= 4 animals in dexamethasone treated group and 6 control animals; * *P *< 0.05 dexamethasone treatment versus control group; # *P *< 0.05 week 20 versus week 15 in control group). ^18^F-FDG, 2-[^18^F]fluoro-2-deoxy-D-glucose; PET, positron emission tomography.

Inflammation in the spontaneous arthritis model in DBA/1 mice has been described previously as short-lived and presenting as enthesitis or dactylitis. The process is characterized by infiltration of mostly neutrophils and to a lesser extent mononuclear inflammatory cells [13]. To quantify inflammation in the paws in a dynamic way, ten mice (four dexamethasone- and six placebo-treated) were injected with ^18^F-FDG and uptake was measured by PET-scan (Figure 3A). ^18^F-FDG has been shown to accumulate in activated inflammatory cells (neutrophils and macrophages) and serves as a marker of inflammation [21]. Mice treated with dexamethasone showed significantly lower tracer uptake at 15 weeks as compared to controls. By week 20 uptake of the tracer was similar in both groups (Figure 3B) and lower than at 15 weeks.

### Presence of chemokines in SpAD

**Figure 4:**
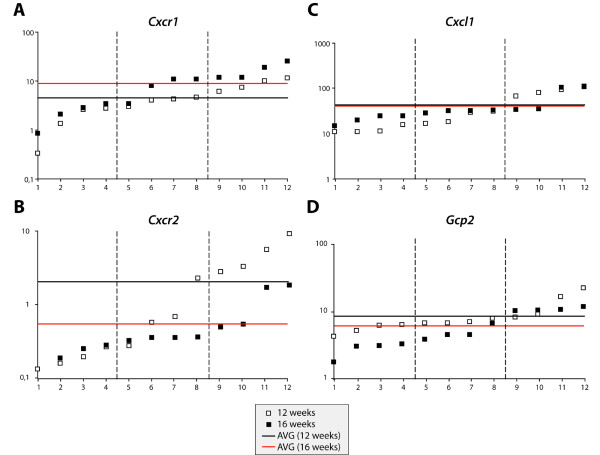
**Quantitative PCR analysis of neutrophil chemokines and receptors in paws of male DBA/1 mice aged 12 and 16 weeks**. The expression levels of *Cxcr1 *(**A**), *Cxcr2 *(**B**), *Cxcl1 *(**C**) and *Gcp2 *(**D**) are shown. Y-axis displays gene expression values normalized to GAPDH and is presented as log-scale (* 10^-4^). The average gene expression for each gene in both groups is presented by the horizontal line. PCR, polymerase chain reaction.

As neutrophils are the most common cells in the inflammatory reaction in this mouse model, we measured expression of two chemokines (*Cxcl1 *and *Gcp2*) and their receptors (*Cxcr1 *and *Cxcr2*), well-known mediators of neutrophil migration to inflammatory sites, in male DBA/1 mice at different time points (Figure 4A-D). Both chemokines and their receptors are expressed in paws of DBA/1 mice, with a proportion of the paws showing high gene expression levels. The fold difference in gene expression level of *Cxcr1*, *Cxcr2*, *Cxcl1 *and *Gcp2 *between the four paws with the highest level compared to the four lowest levels was 5.0, 27.7, 7.2 and 2.5, respectively at 12 weeks and 7.4, 6.3, 3.4 and 3.9 at 16 weeks. Of note, clear clinical signs of disease in these mice were not apparent corroborating our previous observations that mild inflammatory changes in this model are not easily detected by clinical observation only.

### BMPs and chemokine upregulation

**Figure 5:**
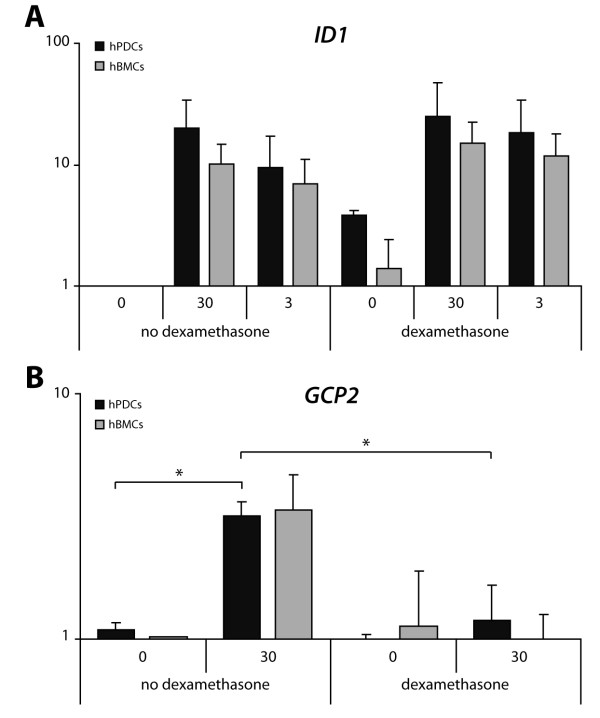
**Gene expression analyses of *ID1 *and chemokines in human periosteal and bone marrow stromal cells**. Cells were cultured for 24 hours in the presence or absence of BMP2 (3 or 30 ng/ml) and 1 μM dexamethasone. Y-axis displays gene expression levels of (**A**) *ID1 *and (**B**) *GCP2 *normalized to housekeeping gene *GAPDH*. Values are expressed as fold increase relative to control condition (no BMP2, no dexamethasone) and are presented as log scale (*n *= 3; * *P *< 0.05). BMP2, bone morphogenetic protein 2.

The entheseal stress hypothesis suggests that activation of entheseal cells may be the initiating event in attracting inflammatory cells [6]. Since earlier observation showed that inhibition of BMP signaling in this model prevented disease occurrence [15], we tested whether BMP2 could induce neutrophil chemokines. Human progenitor cells derived from either periost or bone marrow were stimulated with different concentrations of BMP2 and treated with 1 μM dexamethasone. Stimulation of hPDCs and hBMCs resulted in a dose dependent increase of *ID1*, a BMP target gene, and was not influenced by dexamethasone treatment (Figure 5A). Twenty-four hours of BMP2 stimulation induced the expression of *GCP2 *in hPDCs and this induction was inhibited by dexamethasone (Figure 5B). A similar effect was suggested in hBMCs, although the difference was not statistically significant. *CXCL1 *was also significantly upregulated in hPDCs but not in hBMCs (data not shown). Dexamethasone treatment itself induced an increase in *CXCL1 in vitro *in both hPDCs and hBMCs, but did not further stimulate or inhibit the effect of BMP2.

## Discussion

Patients who are suffering from AS show a remarkable paradox in the skeleton. Inflammation appears associated with osteoporosis of the trabecular bone, but also with new bone formation occurring at the edges of the bone, two contrasting features in close proximity. Targeted therapies antagonizing TNF result in substantial clinical improvement, reduction of inflammation and inhibition of bone loss in AS patients. In contrast, new bone formation leading to ankylosis does not appear to be inhibited by anti-TNF therapy [7-10]. The entheseal stress hypothesis challenges the paradigm that inflammation triggers ankylosis and proposes a concept in which both inflammation and new tissue formation in AS are secondary to a common trigger, for example, cell stress or microdamage in the enthesis [6]. In the study presented here, glucocorticoid treatment in mice developing spontaneous arthritis characterized by ankylosing enthesitis appears to affect inflammation without direct impact on joint remodeling. These observations parallel our earlier studies in which we demonstrated that neither anti-TNF therapy with etanercept nor osteoclast inhibition has a beneficial effect on progression of ankylosis [7,14].

Glucocorticoid use does have a negative effect on bone loss as demonstrated by DEXA analysis. Much like what is seen in AS patients, loss and gain of bone appear simultaneously and in close proximity. This observation provides support for the view that the molecular mechanisms of ankylosis are disconnected from inflammation or bone loss. These observations suggest that new bone formation as seen in this model and also in patients with ankylosing spondylitis is not dependent on the classical bone remodeling cycle essential for skeletal renewal and which is affected in osteoporosis. Therefore, molecular mechanisms leading to new bone formation and ankylosis are likely different from those guiding the physiological bone remodeling cycle. Such a difference suggests that specific targeting of ankylosis is feasible without necessarily interfering with homeostatic bone remodeling.

We previously demonstrated that inhibition of BMP signaling reduced the incidence and severity of spontaneous arthritis and ankylosis suggesting that BMP signals are an upstream event in this mouse model [15]. Inflammation in the spontaneous model is short-lived and the clinical evaluation of the mice does not distinguish between local joint swelling and redness caused by either inflammation, cell proliferation or differentiation [13]. In our experience, the inflammation phase of the model is often discrete and not readily recognized during routine clinical examination of the mice. We, therefore, used ^18^F-FDG PET scans to quantify inflammation in the model. The lack of chronic inflammation is an intrinsic limitation of this model, but the current observation that inhibition of inflammation has no effect on ankylosis corroborates our hypothesis that inflammation and new tissue formation are largely independent events with a potential common trigger.

Further experiments identified upregulation of neutrophil chemokines in the early phase of the model. Since BMP inhibition was, in contrast to different anti-inflammatory strategies, capable of preventing clinical and histomorphological signs of arthritis [15], we further studied whether stromal cells could respond to BMPs by upregulating specific chemokines. BMP-induced GCP2 expression occurred late after stimulation. No significant GCP2 induction could be detected after an early time-point (2-hour stimulation) as measured by gene expression analysis and ELISA by determination of GCP2 protein in the cell culture supernatant (data not shown). Also, dexamethasone treatment did not affect BMP2 induced ID1 expression. Together, these results suggest that the BMP-induced GCP2 expression is an indirect effect.

**Figure 6:**
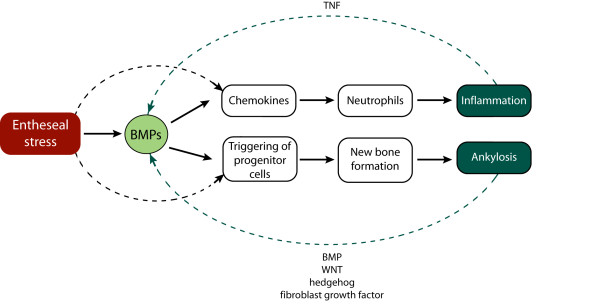
**A model for the dual role of BMPs in the entheseal stress hypothesis**. As a reaction to local stress (damage, strain, and so on} BMP signaling can contribute to inflammation and ankylosis. Joint inflammation in the DBA/1 model is characterized by the presence of neutrophils which are attracted by BMP-induced chemokines. BMP-triggering of entheseal progenitor cells leads to new tissue formation and ankylosis in a complex cascade with multiple feedback mechanisms involving different molecular cascades. Pro-inflammatory cytokines such as TNF are known to stimulate BMPs in mesenchymal cell types and could provide another feedback system. Entheseal cell stress may also trigger other cascades leading to inflammation and tissue differentiation. BMP, bone morphogenetic protein; TNF, tumor necrosis factor.

Our data suggest that at least in this model activation of the BMP signaling cascade can contribute to both joint inflammation and remodeling (Figure 6). These observations further corroborate the entheseal stress hypothesis and provide support for a non-inflammatory or non-specific primary cause of the arthritis with subsequent involvement of both inflammation and remodeling. In the specific entheseal micro-environment complex feedback loops may exist in which TNF and other cytokines stimulate the production BMP2. The BMP2 promoter contains TNF responsive elements and upregulation of BMP2 after TNF stimulation has been demonstrated in different skeletal cell types [22,23].

Taking into account the broad immune-modulating properties of glucocorticoids, we can not assume that the presented interaction with BMP-induced chemokine induction is the only or the main mechanism by which dexamethasone treatment affects the inflammatory disease of the mouse model. Nevertheless, the observed gene regulation provides an exciting link between signaling pathways involved in joint remodeling and signals contributing to inflammation. Such interactions are of great interest when a comprehensive or system-biology view of arthritis is developed.

A number of limitations of the current study need to be considered. First, we preferred to use human progenitor cells instead of mouse cells. Dissection and isolation procedures in the mouse can affect cell purity and it is also difficult to expand mouse periost derived cells into large amounts. hPDCs are periosteum derived progenitor cells involved in bone development and postnatal repair processes [19,24]. The periosteum, a fibrous tissue which covers the bone surface, is known to participate in the differentiation of chondrocytes and osteoblasts in fracture healing [25]. Progenitor cells derived from the bone marrow can be differentiated into osteoblasts and participate as well in bone fracture repair processes [26].

We preferred a broad non-specific anti-inflammatory approach with glucocorticoids to target inflammation *in vivo *rather than the inhibition of a specific chemokine as the inflammatory events are likely driven by different chemokines. A number of small molecules designed to inhibit chemokines are in clinical development but the short-lived pharmacokinetics of such molecules [27,28] are not suitable for the mouse model used here as we demonstrated earlier for p38 inhibition [29] and as suggested by human data using celecoxib in ankylosing spondylitis [30]. Glucocorticoid treatment allowed us to evaluate its effect on local bone formation and systemic bone loss simultaneously.

## Conclusions

This study highlights the paradoxical simultaneous loss and gain of bone in a mouse model of AS and suggests that BMPs may trigger both inflammation and ankylosis. Further identification of factors that lead to the production of BMPs in the enthesis appears, therefore, critical to understand better the initiating events of the disease progress. Insights into these mechanisms based on functional and genomics studies may lead not only to the development of new therapeutic approaches to control and improve AS and related disorders but also to avoid radiographic progression.

## Abbreviations

AS: ankylosing spondylitis; BMP: bone morphogenetic protein; CXCL1: chemokine (C-X-C motif) ligand 1; CXCR1: chemokine (C-X-C motif) receptor 1; CXCR2: chemokine (C-X-C motif) receptor 2; DEXA: dual energy X-ray absorptiometry; ELISA: enzyme-linked immunosorbent assay; ^18^F-FDG: 2-[^18^F]fluoro-2-deoxy-D-glucose; GAPDH: glyceraldehyde-3-phosphate dehydrogenase; GCP2: granulocyte chemotactic protein 2; hBMCs: human bone marrow cells; hPDCs: human periosteum-derived cells; ID1: inhibitor of DNA binding 1; PBS: phosphate buffered saline; PET: positron emission tomography; RT-PCR: reverse transcriptase-polymerase chain reaction; SpA: spondyloarthritides; TNF: tumor necrosis factor.

## Competing interests

KU Leuven holds a patent on behalf of FL and RL on the use of noggin for the treatment of spondyloarthritis. The other authors declare that they have no competing interests.

## Authors' contributions

KB, FL and RL participated in the design of the study. Experiments were performed by KB. CMD participated in the analysis of PET scan data. The manuscript was drafted by KB and was commented on and revised by RL, FL and CMD. All authors read and approved the final manuscript for publication.
